# Introduction and reflections on the comparative physiology of sleep and circadian rhythms

**DOI:** 10.1007/s00360-024-01567-z

**Published:** 2024-06-10

**Authors:** Russell G. Foster

**Affiliations:** Sir Jules Thorn Sleep and Circadian Neuroscience Institute (SCNi), Nuffield Department of Clinical Neurosciences, University of Oxford, Dorothy Crowfoot Hodgkin Building, South Parks Road, Oxford, OX1 3QU UK

**Keywords:** 11-*cis* retinaldehyde, Circadian, Eye, Extraretinal photoreceptors, Opsin, Pineal, Photoreceptors, Sleep

## Abstract

Circadian rhythms and the sleep/wake cycle allows us, and most life on Earth, to function optimally in a dynamic world, adjusting all aspects of biology to the varied and complex demands imposed by the 24-hour rotation of the Earth upon its axis. A key element in understanding these rhythms, and the success of the field in general, has been because researchers have adopted a comparative approach. Across all taxa, fundamental questions relating to the generation and regulation of sleep and circadian rhythms have been address using biochemical, molecular, cellular, system and computer modelling techniques. Furthermore, findings have been placed into an ecological and evolutionary context. By addressing both the “How” – mechanistic, and “Why” – evolutionary questions in parallel, the field has achieved remarkable successes, including how circadian rhythms are generated and regulated by light. Yet many key questions remain. In this special issue on the Comparative Physiology of Sleep and Circadian Rhythms, celebrating the 100th anniversary of the Journal of Comparative Physiology, important new discoveries are detailed. These findings illustrate the power of comparative physiology to address novel questions and demonstrate that sleep and circadian physiology are embedded within the biological framework of an organism.

I am both honoured and delighted to provide some introductory remarks for this Special Issue on the “Comparative Physiology of Sleep and Circadian Rhythms” to celebrate the 100th anniversary of the Journal of Comparative Physiology (JCP). I was asked to provide an introduction that provided some personal reflections regarding my career publications with the JCP. However, before I do this, I would like to consider briefly the importance of the comparative approach for the study of biological mechanisms, and why so many of us in the field of circadian rhythms and sleep have considered the JCP as a natural “home” for the work we do.

Karl Ritter von Frisch (1886–1982) and Alfred Kühn (1885–1968) established the JCP, or as it was called then *Zeitschrift für Vergleichende Physiologie*, in 1924. The JCP has pioneered the publication of articles that study a diverse range of physiological mechanisms, providing breadth beyond just rodent models and human subjects. Articles highlight the similarities and differences in the ways in which animals are physiologically and behaviourally adapted to the varied and complex demands of the environment. From its beginning, and in both laboratory and field studies, the Journal has embraced different experimental approaches that utilise biochemical, molecular, cellular, system and computer modelling techniques. Critically, findings are invariably considered within and broader ecological and evolutionary context of the species. In short, both the “How” – mechanistic, and “Why” – evolutionary questions are considered by the JCP and across the diversity of animal life.

Studies in comparative physiology invariably arise from researchers with curiosity driven questions. However, the findings frequently provide the substrate for translation across the applied sciences. Indeed, the comparative analysis of sleep and circadian rhythms is a perfect example of how fundamental insights have been, and are being, translated into improved health and wellbeing in medicine, agriculture, ecology, biodiversity and environmental science. The early pioneers of the field of circadian rhythms, notably Colin Pittendrigh (Menaker 1996), Jürgen Aschoff (Daan and Gwinner 1998), and Michael Menaker (Foster 2021), were all curiosity driven comparative physiologists. But the field they ignited has led to an extraordinary and expanding translation of this fundamental knowledge. For example, the Nobel Prize in Physiology or Medicine in 2017 (Callaway and Ledford 2017) was awarded for the elucidation of the molecular mechanisms of circadian rhythm generation. This is still the best example we have of how gene expression underpins complex behaviour, and is informing approaches to understand signalling pathways in diverse physiological systems linked to both human and animal health (Fagiani et al. 2022); Fundamental knowledge has led to the foundation of new branches of medicine including, chronometabolism (Yong et al. 2023) and chronopharmacology (Fujimura and Ushijima 2023), and new treatments to regulate sleep and circadian rhythm disruption across the health spectrum, including mental illness (Foster 2022; Freeman et al. 2017). Notwithstanding these successes, very many key questions remain. Not least a fundamental understanding of how sleep is generated and regulated, and why sleep may have evolved in the first place (Foster 2018). In addition, we are only just beginning to understand the mechanisms whereby the sleep and circadian systems interact (Jagannath et al. 2021).

The papers in this special issue address some of these important and unresolved questions. Using a range of different techniques and species, and combined with both laboratory and field studies, exciting new findings are discussed in this Special Issue. Topics range across the seasonal variation in sleep time of jackdaws; Fur seal performance after sleep deprivation; Ultrasonic vocalisation and diurnal activity in Djungarian hamsters; Mechanisms of sleep pressure in zebrafish; and Synaptic plasticity deficits in human neurodevelopmental disorders. These studies capture perfectly the vision and philosophy of the JCP as originally envisaged by von Frisch and Kühn 100 years ago, and it is my sincerest hope that you will enjoy reading these papers as much as I have. I also hope that this new knowledge will inform how you approach your own research.

Now turning to my association with JCP. Allow me to start with a little background. Vertebrates possess two functionally distinct classes of photoreceptors: visual photoreceptors, which capture light to construct an image of the environment and non-visual photoreceptors, which use changes in environmental irradiance to regulate a range of physiological responses, including circadian and sleep physiology (Foster and Soni 1998). Vertebrates have photoreceptors located in a number of different regions of the body. Those arising from the diencephalic forebrain are classified as: (i) intracranial pineal and parapineal organs; (ii) extracranial “third eyes”, also called frontal organs (frogs) or parietal eyes (lizards); (iii) deep brain photoreceptors; and (iv) lateral eyes (Foster et al. 2020; Foster and Soni 1998; Shand and Foster 1999). In addition to the photoreceptors arising from the diencephalon, vertebrates possess further photoreceptors, including tissue-wide photoreceptors (Moutsaki et al. 2003); skin/dermal photoreceptors (Lythgoe, 1984; Provencio et al. 1998); and photoreceptors located within the iris (Xue et al. 2011) and cornea (Diaz et al. 2020) of the eye. I would like to stress, that the detailed study of these extraretinal photoreceptors was stimulated by the early work of Karl von Frisch.

Karl von Frisch is best known for his work on bees, their colour vision and ability to communicate sources of nectar to other bees using a time-compensated sun-compass “waggle dance” behaviour (Frisch 1993) for which he was awarded the Nobel Prize in 1973 with Konrad Lorenz and Nikolaas Tinbergen. But it was von Frisch who provided the first detailed investigation of a “deep encephalic photoreceptor” in 1911 (Frisch 1911). Von Frisch moved from the study of medicine to zoology, and his PhD work explored the light induced colour changes in the skin of European minnows (*Phoxinus phoxinus*). He showed that blinded and intact minnows became pale in the dark, and then darkened upon exposure to light. When blinded animals also had their pineal removed, the melanophore responses to light were abolished, but returned after one day. Subsequently, von Frisch found that lesions within the diencephalon would completely block this response to light. He concluded that there must be photoreceptive elements within the diencephalon. In 1928 Ernst Scharrer, considered to be the “father” of neurosecretion, built upon these observations and demonstrated that the encephalic photoreceptors of the European minnow also regulated swimming and feeding behaviours. On the basis of these observations, and those made later with amphibians and reptiles, Scharrer proposed that there were “photoneuroendocrine” cells within the basal brain and pineal (Scharrer 1964).

In the 1930’s evidence for deep brain, pineal and dermal photoreception followed in lampreys, with John Zachary (JZ) Young showing that all of these photoreceptor classes are involved in triggered body movements (Young 1935a, b). Also in the 1930’s, and this time in ducks, Jacques Benoit showed that long photoperiod illumination delivered directly to the hypothalamus using glass rods caused testicular growth in blinded mallards (Benoit 1935a, b). In the 1960s, work on lizards and birds by Michael (Mike) Menaker and colleagues demonstrated that the regulation of circadian rhythms and the photoperiodic control of reproduction was mediated by extraretinal photoreceptors (Menaker and Underwood 1976; Oliver et al. 1977; Underwood and Menaker 1976; Yokoyama et al. 1978). Again in the 1960’s, elegant electrophysiological recordings of light responses from the fish and amphibian pineal complex were recorded by Eberhard Dodt and colleagues (Dodt 1963; Dodt and Heerd 1962). As a student of zoology in the late 1970’s I was introduced to these non-visual photoreceptors during lectures, laboratory classes, and background reading (Young 1962) and became utterly fascinated, if not a little obsessed, with these extraordinary photosensory systems. This interest has defined much of my career, and I have published more than nine publications on this topic in the JCP, some of which I discuss in this Introduction.

As an undergraduate at the University of Bristol, UK I attended a seminar by Alan Roberts who described his experiments showing that the embryos and young larval stages of the African Clawed toad (*Xenopus laevis*) would start swimming when exposed to a sudden drop in light intensity, and that the intact pineal eye (frontal organ) was essential for this response (Roberts 1978). During my final year as an undergraduate I was able to investigate Alan’s observations by recording the electrophysiological responses of the pineal eye. Suddenly lowering the light intensity evoked a burst of action potentials, whilst increasing the light intensity led to a lowered firing frequency. Prolonged exposure to white light at a range of different irradiances, showed that spike frequency was dependent upon light intensity and that the pineal eye, at this stage of development at least, can act as an irradiance detector. Exposing the pineal to a range of wavelengths at different irradiances allowed the construction of an action spectrum. These data defined an opsin/vitamin A_2_ based photopigment with a maximum spectral response (λ_max_) at 520 nm. We suggested that this “red” shifted λ_max_ would probably allow enhanced sensitivity, making the pineal eye more sensitive to the longer wavelengths of light that penetrate the freshwater environment (Lythgoe 1979). This work became my first full publication, and was published in the JCP in 1982 (Foster and Roberts 1982). The stage was then set for my PhD, and studies on another group of extraretinal photoreceptors.

As mentioned, Benoit in the 1930’s showed that birds use a photoreceptor located deep within the deep brain to detect daylength changes for the regulation of seasonal (photoperiodic) physiology, but for more than 50 years nothing was known about the nature of this photoreceptor. This was the topic of by PhD supervised by Brian Follett. I developed a novel technique to investigate the photopigment involved in the photoperiodic control of reproduction in Japanese quail (*Coturnix coturnix*). When these deep brain photoreceptors were exposed to “long days” (20 h of light:4 h of dark) of either white or monochromatic light a clear relationship was demonstrated between light irradiance and the release of luteinizing hormone (LH) from the pituitary gland. A quantitative determination of the differential absorption and scatter of light passing through the skull and into the basal brain allowed me to illuminate the hypothalamic region of the brain with equal numbers of photons at a range of wavelengths, allowing the construction of an action spectrum for photoperiodic induction. The results provided the first demonstration, in any vertebrate, that the deep brain photoreceptors are based upon an opsin/vitamin A_1_ photopigment. The peak sensitivity (λ_max_) of the photopigment was shown to be at 492 nm. Interestingly, this peak in sensitivity corresponds to a small “window” in haemoglobin absorption, suggesting that the deep brain photoreceptors are spectrally “tuned” to the dominant wavelengths of light reaching the basal brain. These findings were published in JCP in 1985 (Foster and Follett 1985).

Although the biochemical identity of the deep brain photoreceptor had been defined by the original JCP paper (Foster and Follett 1985), the cellular and molecular identity of these photoreceptors remained a mystery for a further 24 years! We isolated a new photopigment family designated VA opsin, first in fish (Soni and Foster 1997; Soni et al. 1998) and then chickens (Halford et al. 2009). Significantly, we showed that VA opsin is expressed within a population of hypothalamic neurones in the avian brain (Halford et al. 2009), and critically, the original action spectrum published in JCP precisely matched the absorption spectrum for the isolated VA opsin photopigment - at 492 nm (Davies et al. 2012). Finally, we were able to show that VA opsin is co-expressed with Gonadotropin hormone-releasing hormone (GnRH) neurones within the hypothalamus, suggesting that these neuroendocrine cells may possess endogenous photosensitivity (García-Fernández et al. 2015). In this regard these cells represent a perfect example of the “photoneuroendocrine” cells, whose existence was originally proposed by Ernst Scharrer in the 1920’s (Scharrer 1964). Collectively these findings have had a major impact upon our understanding of how day length information is transduced into avian seasonal biology. Multiple questions remain, and work on these deep brain photoreceptors continues (Perez et al. 2023).

At the end of the 1980’s we published two further papers in JCP that explored the photoreceptor capacity of the avian and mammalian pineal. Immunocytochemistry with a rod-specific antiserum was used to study the post-hatch development (2 days–300 days) of photoreceptor elements within the pineal of the Japanese quail. At all ages staining was restricted to limited numbers of pinealocytes scattered throughout the gland. An enzyme-linked immunosorbent assay (ELISA), with the same rod-specific antibody, was then used to obtain a quantitative measure of rod opsin in total eye and pineal extracts. In parallel we looked for the presence of vitamin A photopigment chromophore, 11-*cis* retinaldehyde, within pineal and retinal extracts using HPLC analysis. In both retinal and pineal extracts, 11-*cis* retinaldehyde was identified and a light-induced shift from the 11-*cis* to the all-trans isomer was clearly shown. A functional photopigment requires both an opsin and 11-*cis* retinaldehyde in a ratio of 1:1. Significantly, we found more than double the concentration of 11-*cis* retinaldehyde within the pineal compared to the amount of rod-like opsin. These results, coupled with our immunocytochemical findings, suggested that the quail pineal contains at least two classes of photoreceptor, with some pinealocytes utilising a rod-like opsin, whilst others use one or more unidentified photopigments (Foster et al. 1989a). These studies anticipated the identification of a novel pineal photopigment called “P-Opsin” (Max et al. 1995).

Previously, photopigment “rod-like” opsins had been identified within the mammalian pineal using immunocytochemistry, and this led to the suggestion that the mammalian pineal, like the pineal of other vertebrates, might be photosensitive (Korf et al. 1985). But as mentioned, a functional photopigment requires both an opsin and 11-*cis* retinaldehyde in a ratio of 1:1. We used the same methodology as used for the quail pineal studies to measure opsin levels and 11-*cis* retinaldehyde within the pineal of adult Djungarian hamsters. Whilst we successfully quantified opsin within the hamster pineal using ELISA, we found no evidence of 11-*cis* or all-trans retinaldehyde. These findings were published in JCP, and we were able to conclude that the opsin present within the adult hamster pineal is not coupled to a retinaldehyde chromophore, and as a result, the opsin present is highly unlikely to be part of a functional photopigment (Foster et al. 1989b). Why opsins are expressed within the mammalian pineal remains an intriguing question (Foster et al. 2003).

Using similar methodological approaches, we then focused our attention on the deep brain photoreceptors of the lizard *Anolis carolinensis*. Three different antibodies localised opsins within the hypothalamus and septal region of the lizard brain and Western blot analysis demonstrated that these antibodies recognized a single protein at the correct molecular weight for an opsin of around 40 kD in ocular, anterior brain and pineal extracts. Immunoblots of rodent brain did not identify this protein band. Significantly, 11-*cis* and all-*trans* retinaldehyde were identified within anterior brain extracts using HPLC. Back in 1993, these findings provided the most detailed analysis of a deep brain photoreceptor in any vertebrate, and were published in JCP (Foster et al. 1993).

Having worked on the extra-retinal photoreceptors in non-mammalian vertebrates, I became interested in how the circadian rhythms of mammals are regulated (entrained) by light. It was clear that mammals required their eyes for entrainment as enucleation or covering the eyes blocked any ability to entrain. At the time it was assumed that the rods and cones were used for both image-forming and non-image forming (irradiance) light detection. But I was uneasy with this assumption. I simply could not understand how the rods and cones, so exquisitely evolved to generate an image of the world, could also be used to extract time-of-day information. For vision to work, the retina has to grab light, and then in a fraction of a second later, forget this light exposure and be ready for the next visual image. If this very rapid “grab and forget” did not occur then our world would not be a series of sharp images, but a constant blur of shades of light, dark and colour. Importantly, a sharp image is not needed for entrainment. Instead, the circadian system requires an impression of the overall amount of light around dawn and dusk. Such broad changes in the light environment occur over minutes and hours. So, my simple question was “how can the eyes be used for both the sensory tasks of vision and the regulation of biological time?”

With this question in mind, we decided to look at the circadian responses to light of mice with hereditary retinal disorders. Our first mouse model was the *retinal degeneration* or *rd* mouse. Mice homozygous for the *rd* mutation (*rd/rd*), lack functional rods and possess a limited number of highly degenerate cones. These mice fail to show any visually mediated behaviours, and are functionally blind. The sensitivity of circadian photoreception in these mice was determined by varying the irradiance of a 15 min light pulse (515 nm) given at circadian time (CT) 16 and measuring the magnitude of the phase shift of the locomotor rhythm. Full irradiance response curves were undertaken in visually blind homozygous *rd/rd*, sighted heterozygous *rd/+* and fully sighted wild-type *+/+* mice 80 days of age. Despite the loss of visual photoreceptors, and being functionally blind, *rd/rd* mice showed normal and unattenuated circadian responses to light. We established that the eye mediated this light entrainment as eye loss in these mice blocked these responses. On the basis of our results, published in JCP (Foster et al. 1991), we proposed in 1991 that the eye might contain an unrecognised photoreceptor within the eye responsible for circadian regulation. In a sense, that mammals had an extraretinal photoreceptor within the eye! Because of my background in extraretinal photoreceptors, it did not seem at all unreasonable to make this suggestion. However, this proposal was met by remarkable hostility when presented. On one occasion at a scientific meeting a member of the audience shouted “bullshit”. A criticism of the JCP study was that the few degenerate cone photoreceptors might be sufficient for normal circadian responses to light. This was theoretically very unlikely, but our first study in JCP could not preclude this possibility. Our response was to develop two genetically-engineered mouse models that entirely lacked their rods and cones (*rd/rd cl* and *Rdta/cl*). These models allowed us to show that a broad range of responses to light including circadian entrainment (Freedman et al. 1999), melatonin synthesis (Lucas et al. 1999) and pupil constriction (Lucas et al. 2001a) occur in the absence of rod and cone photoreceptors. By 1999, our two back-to-back *Science* papers, demonstrated unequivocally that the mouse eye must contain a third class of photoreceptor, and these studies provided the conceptual framework for the identification of a “blue light sensitive” (Lucas et al. 2001b) melanopsin-based photosensitive retinal ganglion cells (pRGCs) in the mouse (Hattar et al. 2003; Sekaran et al. 2003), rat (Berson et al. 2002), Macaque Monkey (Dacey et al. 2005) and human retina (Hannibal et al. 2017; Zaidi et al. 2007). For a full discussion see (Foster et al. 2020).

The first evidence for a non-rod and non-cone ocular photoreceptor was published in JCP (Foster et al. 1991). The then editor of JCP was Hansjochem Autrum, taking over from von Frisch in 1960. I greatly appreciated our discussions and his enthusiastic support of the work. A key point is that our curiosity-driven research is now having a major impact in clinical ophthalmology. Our findings argued that the clinical diagnosis of ‘complete’ blindness should assess the state of both the rod/cone and pRGC photoreceptive systems. We now appreciate that eye loss plunges individuals into a world that lacks both vision and a proper sense of time and we are developing clinical guidelines that incorporate this information. For example, if a “blind” individual shows a bright light-dependent pupil constriction, which we showed is dependent upon the pRGCs (Zaidi et al. 2007), then they should be encouraged to expose their eyes to sufficient day-time light to maintain normal circadian entrainment and sleep-wake timing. Furthermore, patients with diseases of the inner retina which result in retinal ganglion cell death, such as glaucoma, are at particular risk of circadian rhythm and sleep disruption. We have published a series of papers on the impact of multiple types of eye disease on sleep/wake timing in humans e.g. (Alexander et al. 2014; Andrews et al. 2019; Cuthbertson et al. 2009; Morjaria et al. 2019), and such studies are forming the basis of evidence-based guidelines for clinical ophthalmology (Douglas and Foster 2015). And finally, our most recent work has been the dissection of the light signalling pathway from the pRGCs to the molecular clockwork (Jagannath et al. 2013, 2021, 2022). Once again, this curiosity driven research, with its origins in the JCP paper (Foster et al. 1991), is being translated to develop new drugs that “mimic” the effects of light on the circadian system. Our hope is that we will be able to give back a sense of time to the “time blind”, not least, in those individuals suffering radical eye damage or eye loss.

The Journal of Comparative Physiology has pioneered the comparative approach to research for 100 years, and the Journal’s contribution to supporting fundamental knowledge has been extraordinary. The field of circadian rhythms and sleep has a long and special relationship with the JCP, as exemplified by this Special Issue on the “Comparative Physiology of Sleep and Circadian Rhythms”. Many circadian and sleep researchers have published in the JCP, and I hope this tradition will continue. Indeed, I am sure that as long as we continue to generate first-class science JCP will be eager to consider it for publication – I hope!
